# Destabilisation of dimeric 14-3-3 proteins as a novel approach to anti-cancer therapeutics

**DOI:** 10.18632/oncotarget.3995

**Published:** 2015-05-04

**Authors:** Joanna M. Woodcock, Carl Coolen, Katy L. Goodwin, Dong Jae Baek, Robert Bittman, Michael S. Samuel, Stuart M. Pitson, Angel F. Lopez

**Affiliations:** ^1^ Centre for Cancer Biology, SA Pathology and University of South Australia, Adelaide, SA, Australia; ^2^ Department of Chemistry and Biochemistry, Queens College of the City University of New York, Flushing, NY, USA; ^3^ School of Medicine, Faculty of Health Sciences, University of Adelaide, Adelaide, SA, Australia

**Keywords:** biochemistry, signal transduction, sphingosine, apoptosis, small molecules

## Abstract

14-3-3 proteins play a pivotal role in controlling cell proliferation and survival, two commonly dysregulated hallmarks of cancers. 14-3-3 protein expression is enhanced in many human cancers and correlates with more aggressive tumors and poor prognosis, suggesting a role for 14-3-3 proteins in tumorigenesis and/or progression. We showed previously that the dimeric state of 14-3-3 proteins is regulated by the lipid sphingosine, a physiological inducer of apoptosis. As the functions of 14-3-3 proteins are dependent on their dimeric state, this sphingosine-mediated 14-3-3 regulation provides a possible means to target dimeric 14-3-3 for therapeutic effect. However, sphingosine mimics are needed that are not susceptible to sphingolipid metabolism. We show here the identification and optimization of sphingosine mimetics that render dimeric 14-3-3 susceptible to phosphorylation at a site buried in the dimer interface and induce mitochondrial-mediated apoptosis. Two such compounds, RB-011 and RB-012, disrupt 14-3-3 dimers at low micromolar concentrations and induce rapid down-regulation of Raf-MAPK and PI3K-Akt signaling in Jurkat cells. Importantly, both RB-011 and RB-012 induce apoptosis of human A549 lung cancer cells and RB-012, through disruption of MAPK signaling, reduces xenograft growth in mice. Thus, these compounds provide proof-of-principle for this novel 14-3-3-targeting approach for anti-cancer drug discovery.

## INTRODUCTION

The family of 14-3-3 proteins plays a pivotal role in integrating cellular survival signaling and thereby are key players in determining the cell fate [1, 2]. Through their role in binding multiple phospho-client proteins within the cell, 14-3-3 proteins regulate many important signaling events. In particular, 14-3-3 proteins maintain the proliferative capacity of the cell by supporting the efficient activation of the Raf-MAPK signaling cascade and also support cell survival through the PI3K-AKT signaling cascade [1, 2], key requirements of cancer cells [3].

Enhanced expression of 14-3-3 proteins has been detected in many human cancers including lung [4], head and neck [5], breast [6] and ovarian cancer [7] and correlates with more aggressive tumors and poor prognosis [8]. Over-expression of 14-3-3 isoforms has been shown to contribute to neoplastic transformation by stimulating Raf-MAPK and PI3K signaling [9]. Down-regulation of 14-3-3ζ in head and neck cancer cells [10] and also lung cancer cells [11] renders cells more sensitive to chemotherapy, supporting the notion that cancer cells utilize mechanisms that are 14-3-3 dependent. These experimental and clinical observations suggest that 14-3-3 proteins represent an addiction for many cancers and consequently are an attractive target for anti-cancer therapy [8, 12].

In search of 14-3-3 inhibitors, several studies have identified small molecules that function as mimics of 14-3-3 binding partners, blocking the interaction of 14-3-3 proteins with phospho-clients such as c-Abl [13] and Raf-1 [14], and thus behaving as competitive antagonists. These small molecule inhibitors of 14-3-3 proteins exhibit anti-cancer activity in cell-based assays but are limited by their specificity, ability to penetrate cells and also the need for high concentrations to effectively compete with abundant endogenous 14-3-3-binding proteins.

We have taken a novel approach to developing 14-3-3-targeting molecules by exploiting the requirement of 14-3-3 proteins to function as dimers [15]. 14-3-3 proteins are intrinsically dimeric in nature, a characteristic which is obligatory for many of their biological functions [16]. We showed previously that the dimeric status of 14-3-3 proteins is subject to regulation, with the critical step being phosphorylation of Ser58 (numbering relates to the ζ isoform), a site otherwise buried in the dimer interface [17]. The Ser58 dimer interface phosphorylation site is conserved in five of the seven mammalian isoforms and is recognized by several kinases [18-20] but, importantly, we showed it only becomes accessible after the endogenous lipid sphingosine binds to the 14-3-3 protein [15]. Once Ser58 is phosphorylated, the dimeric structure of the 14-3-3 protein is disrupted and its function is inhibited [17]. Thus, through this mechanism, sphingosine serves as a key regulator of dimeric 14-3-3 protein function and induces apoptosis [15].

Similarly, we found that the synthetic sphingosine analogue, FTY720, also renders 14-3-3 phosphorylatable [15]. FTY720, also known as Fingolimod and Gilenya^TM^, is in clinical use to induce immune suppression in the treatment of multiple sclerosis, but has reported anti-cancer characteristics in many experimental systems [21]. As an immune suppressant, FTY720 is a pro-drug which relies on conversion to a phosphorylated form for its effects, predominantly as a sphingosine-1-phosphate receptor antagonist [21]. The anti-cancer effects of FTY720 however are associated with the unphosphorylated pro-drug form [21] and we have shown that these are mediated in part by its effect on 14-3-3 proteins [15]. Owing to its immunosuppressant properties, FTY720 is not a suitable anti-cancer therapy but understanding the molecular basis of its anti-cancer action has provided us with a rationale to identify more sphingosine-like compounds that target 14-3-3 specifically.

We have now surveyed other sphingosine-like molecules and demonstrate that *N*-alkylated trimethyl ammonium (TMA) molecules act as sphingo-mimics to disrupt 14-3-3 dimers and induce apoptosis in Jurkat cells. Because these compounds are unsuitable drug candidates, we have combined the chemical nature of the TMAs with the FTY720 backbone to generate a novel chemical series that exhibit apoptotic characteristics through a 14-3-3-mediated mechanism *in vitro*. Furthermore, we have demonstrated that the most potent of this chemical series induce mitochondrial-mediated apoptosis at low micromolar concentrations *in vitro* and rapidly elicit a signaling cascade that corresponds kinetically to the disruption of dimeric 14-3-3 functions. In a mouse xenograft model of human non-small cell lung cancer where 14-3-3 is over-expressed, RB-012, our most active compound significantly reduced tumor growth without adverse effects on the animals. Our data show that RB-012 is soluble, readily taken up by cells, effective at low concentration and unlike other previously reported 14-3-3-directed small molecules, acts via a non-competitive mechanism. This compound and the mechanism that underlies its activity provide proof-of-principle for our approach to developing a new class of 14-3-3-targeting small molecule therapeutics for cancer treatment.

## RESULTS

### Trimethylammonium compounds render the 14-3-3 dimer interface accessible to kinases and induce mitochondrial apoptosis

**Figure 1:**
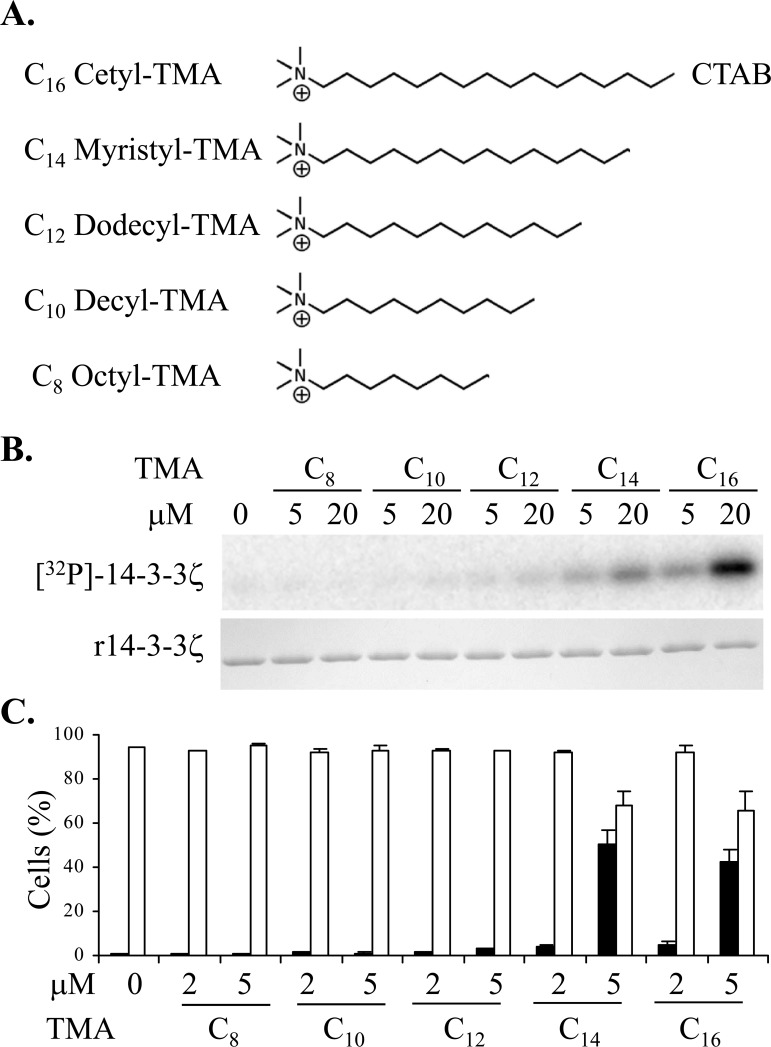
**A.** Structures of the trimethylammonium (TMA) compounds assessed for 14-3-3 modulating activity. **B.** Phosphorylation of 14-3-3 by PKA *in vitro* in presence or absence of TMA compounds at the concentrations shown. The upper panel is [^32^P]-phospho-labeled 14-3-3ζ and the lower panel is Coomassie stained 14-3-3 protein. **C.** Effect of TMA compounds on Jurkat cell after 20 h treatment at the concentrations shown. Cell viability is shown in open bars and TMRE negative staining cells are shown in black bars. The error bars show the range of duplicate determinations: and the results are representative of multiple experiments.

We previously established that non-acylated sphingolipids with a net positive charge are capable of rendering 14-3-3 phosphorylatable [15]. To identify new compounds that are capable of rendering 14-3-3 phosphorylatable but are not susceptible to sphingolipid metabolism, we assessed non-sphingoid cationic lipids such as quaternary ammonium compounds for effects on 14-3-3 phosphorylatability. In our *in vitro* system using recombinant 14-3-3ζ as substrate and PKA catalytic subunit as the phosphorylating enzyme, we found that trimethylammonium (TMA) molecules with an alkyl chain of 14 carbons or longer rendered 14-3-3 phosphorylatable, whereas molecules with a shorter alkyl chain were ineffective (Figure 1A & 1B).

We next tested whether the *N*-alkyl TMA series could induce cell death of Jurkat cells. Cell death was assessed using flow cytometry after 20 h of treatment by analysis of cell viability together with tetramethylrhodamine ethyl ester (TMRE) staining to monitor mitochondrial permeability transition (ΔΨ_M_), an event commonly associated with programmed cell death (Figure 1C). The longer chain *N*-alkyl TMA molecules induced mitochondrial permeability transition at 5μM, consistent with their ability to render 14-3-3 phosphorylatable. This suggests that like sphingosine, these longer chain TMA molecules can regulate dimeric 14-3-3 proteins to disrupt their functions in cells.

**Figure 2:**
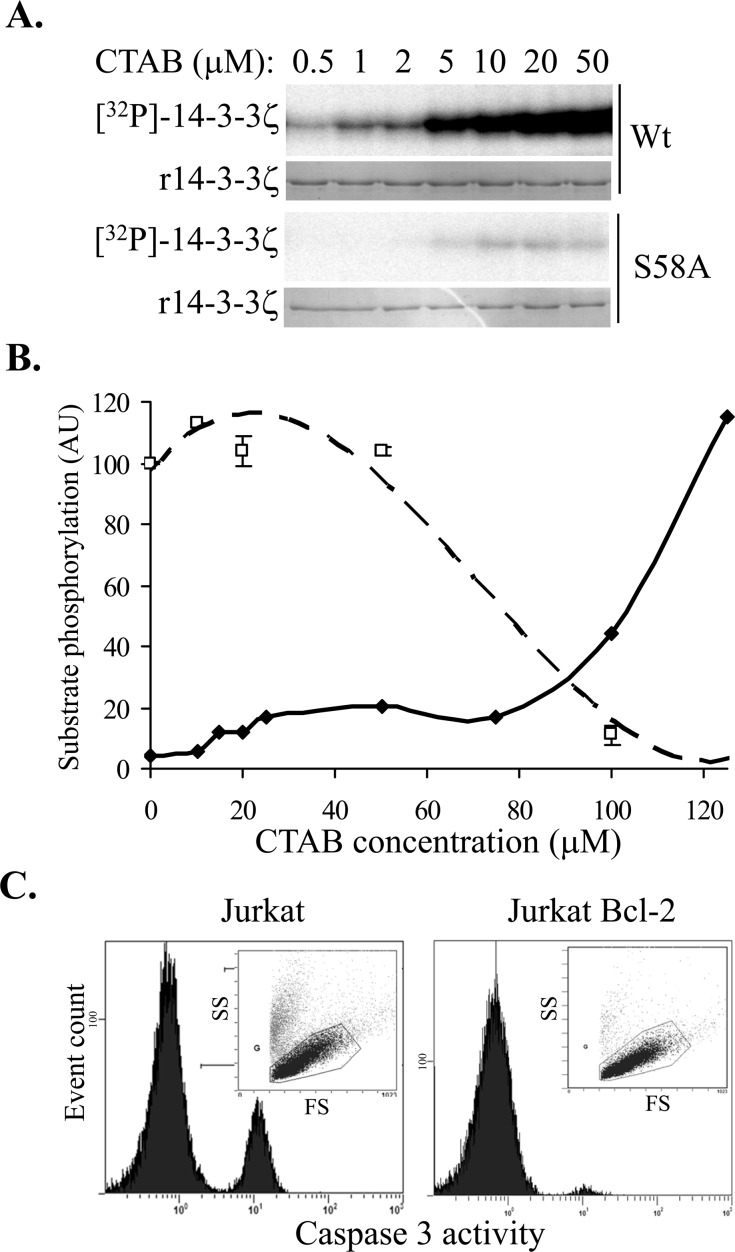
**A**. *In vitro* phosphorylation of 14-3-3ζ (Wt and S58A) by PKA in the presence of increasing concentrations of CTAB (C_16_-TMA). **B.** Quantitation of 14-3-3ζ phosphorylation (solid symbols and solid line) and PKA activity (open symbols and dashed line) with increasing CTAB concentration. **C.** Effect of 5μM CTAB on cell viability (FS *vs*. SS plots inset) and caspase-3 activation (histograms) in parental Jurkat cells (left panel) and Jurkat cells over-expressing Bcl-2 (right panel) after 20 h.

To elucidate the mechanism of the TMA compound's effect on 14-3-3 protein, we carried out dose-response studies with C_16_-TMA, (cetyltrimethylammonium bromide, denoted as CTAB) in 14-3-3 phosphorylation reactions. We observed a dose-dependent increase in 14-3-3 Ser58 phosphorylation with increasing concentration of CTAB (Figure 2A) and using recombinant S58A 14-3-3ζ protein, confirmed that Ser58 was the only site phosphorylated by PKA (Figure 2A), consistent with our previous studies with sphingosine, and FTY720 [15]. Additionally, we observed no effect of CTAB on PKA catalytic subunit activity (as determined using kemptide phosphorylation) over the same concentration range (data not shown), reinforcing that CTAB has a direct effect on the 14-3-3 protein, not on enzyme activity.

Long chain *N*-alkylated TMA molecules are amphiphilic with a cationic head group and a long alkyl chain and can therefore behave as cationic detergents. The reported critical micelle concentration for CTAB in water is 1 mM but in 20 mM Tris-Cl pH 7.0, 10 mM NaCl the CMC decreases to ~ 0.15 mM [25]. We therefore assessed CTAB's effect on 14-3-3 phosphorylation more closely to determine whether non-specific denaturation of 14-3-3 was contributing to the phosphorylatability of 14-3-3 by PKA. In extended dose-response studies, CTAB induced 14-3-3 phosphorylation at low micromolar concentrations, plateauing at 25 μM, but above 75 μM a further increase in 14-3-3 phosphorylation was observed (Figure 2B). This implies that at low micromolar concentrations, CTAB binds discrete sites in 14-3-3, thereby conformationally altering the dimer interface to reveal the Ser58 phosphorylation site accessible to PKA, whereas at higher concentrations of CTAB, approaching CMC values, a more cooperative effect is seen, consistent with generalized protein denaturation. Consistent with this, PKA catalytic subunit activity was significantly inhibited at higher CTAB concentrations (Figure 2B, kemptide phosphorylation was reduced by 90% in the presence of 100 μM CTAB compared with vehicle alone) presumably due to the denaturation of the enzyme. Similar effects have been observed for C_14_-TMA binding to bovine serum albumin, with saturable binding to specific high affinity sites at low C_14_-TMA concentrations and cooperative non-specific binding at high concentrations [26].

To test whether the mitochondrial permeability transition induced by CTAB is associated with apoptosis, akin to the effect of sphingosine and FTY720 [15], we examined caspase-3 activation, a characteristic marker of apoptosis. As shown in Figure 2C, CTAB does induce caspase-3 activation in Jurkat cells and, as with FTY720 [27], Jurkat cells over-expressing Bcl-2 were protected from CTAB-induced cell death, confirming that the CTAB-induced apoptosis is mediated by the mitochondria. Thus, these data show that at concentrations well below the CMC, *N-*alkyl TMA molecules are able to bind to 14-3-3 proteins in cells, leading to disruption of 14-3-3 dimers and subsequent induction of mitochondrial apoptosis.

### Development of the novel RB sphingomimetics and their modulation of 14-3-3 dimerisation and induction of mitochondrial apoptosis

**Figure 3:**
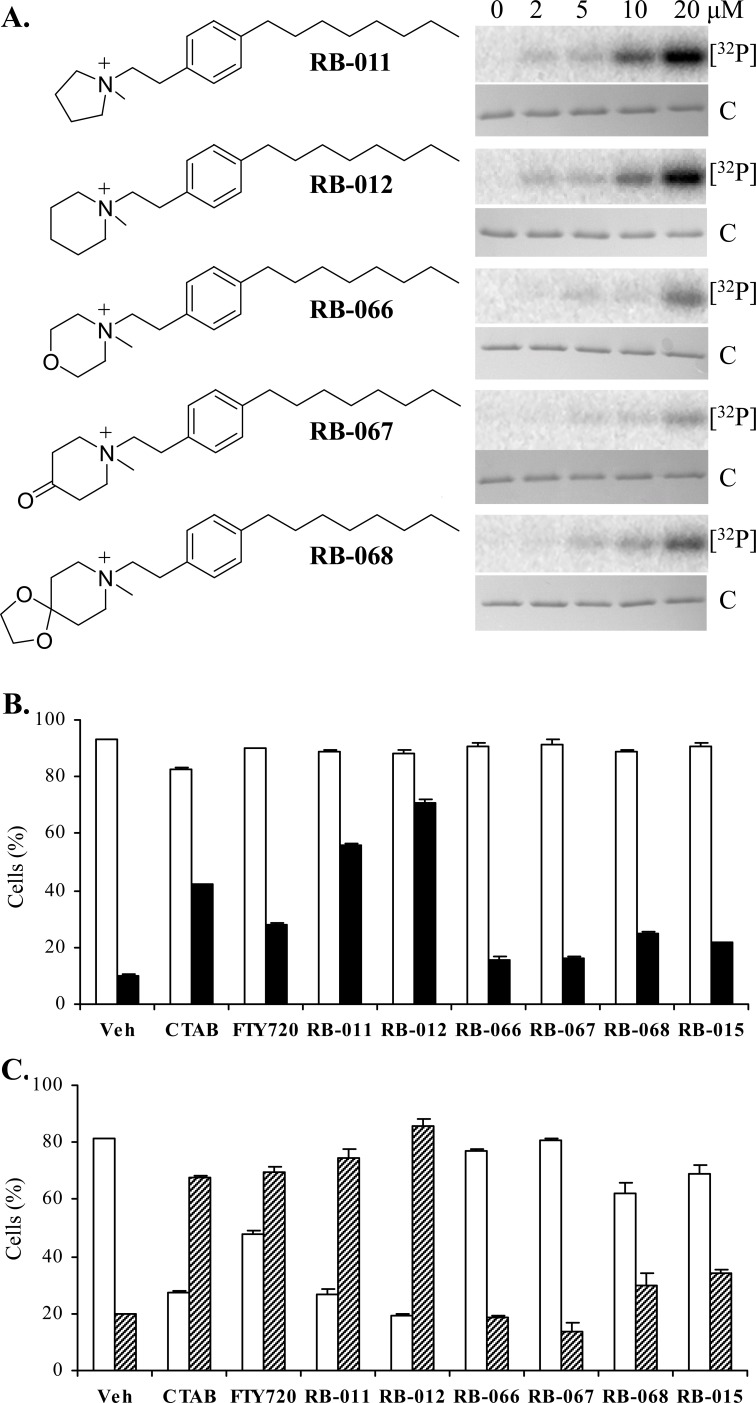
**A.** Structures of the TMA-FTY720 hybrid RB compounds and their effect on *in vitro* 14-3-3ζ phosphorylation by PKA at the concentrations as shown. The upper panel is [^32^P]-phospho-labeled 14-3-3ζ ([^32^P]) and the lower panel Coomassie-stained 14-3-3ζ protein (C). **B.** Effect of 5 μM RB molecules on viability (open bars) and caspase-3 activation (black) of Jurkat cells after 5 h treatment. **C.** Effect of 5 μM RB compounds on viability (open bars) and Annexin V staining (hashed bars) of Jurkat cells after 24 h treatment. The error bars show the range of duplicate determinations and the results are representative of multiple experiments.

Our results with CTAB suggest that at concentrations below its CMC, this molecule can bind to discrete sites in 14-3-3 proteins, causing conformational changes at the dimer interface and thereby allowing kinases access to the Ser58 phosphorylation site. CTAB however, is not an ideal anti-cancer candidate owing to its strong detergent properties and known toxic effects in mice. Data generated by the Developmental Therapeutics Program at the National Cancer Institute indicate that CTAB is toxic in mice when dosed above 10 mg/kg over a five day treatment, (mined from <http://dtp.nci.nih.gov/dtpstandard/dwindex/index.jsp>). The clinical drug FTY720 has previously been used in mice at 10 mg/kg with no reported adverse effects, although its clinical application is as an immunosuppressant, an activity associated with the phosphorylated form of FTY720 [21]. In order to generate more 14-3-3-selective agents we sought to combine the charged quaternary ammonium group of the TMA molecules with the clinically approved FTY720 alkyl chain, and thereby generated a panel of new quaternary amine derivatives of FTY720, denoted here as RB-011, -012, -066, -067 and -068 (Figure 3A). Unlike sphingosine and FTY720, these analogues lack hydroxyl sites for phosphorylation by sphingosine kinases and therefore cannot be converted to immunosuppressive phospho-species.

Initially we tested the five RB compounds in our *in vitro* 14-3-3 phosphorylation assay. In dose response studies we found RB-011 and RB-012 were the most effective at rendering 14-3-3 phosphorylatable by PKA, whereas the other compounds were much less effective (Figure 3A). We then assessed the ability of the RB compounds to induce apoptosis of Jurkat cells compared with FTY720, CTAB and RB-015 [22], a hydroxylated version of RB-012 (in which the hydroxyl group is at the 4 position of the piperidinium ring, ref. 22). At 5 μM, RB-011 and -012 were readily able to activate apoptosis as determined by caspase-3 activation within 5 h (Figure 3B), and Annexin V presentation and loss of viability at 24 h (Figure 3C), consistent with their ability to elicit 14-3-3 phosphorylation at low concentration. Importantly RB-011 and -012 were more potent than either CTAB or FTY720. None of the other RB compounds were as effective at inducing Jurkat cell apoptosis consistent with their effect on 14-3-3 phosphorylation and indicating that ring substitution is not tolerated for these activities (Figure 3).

**Figure 4:**
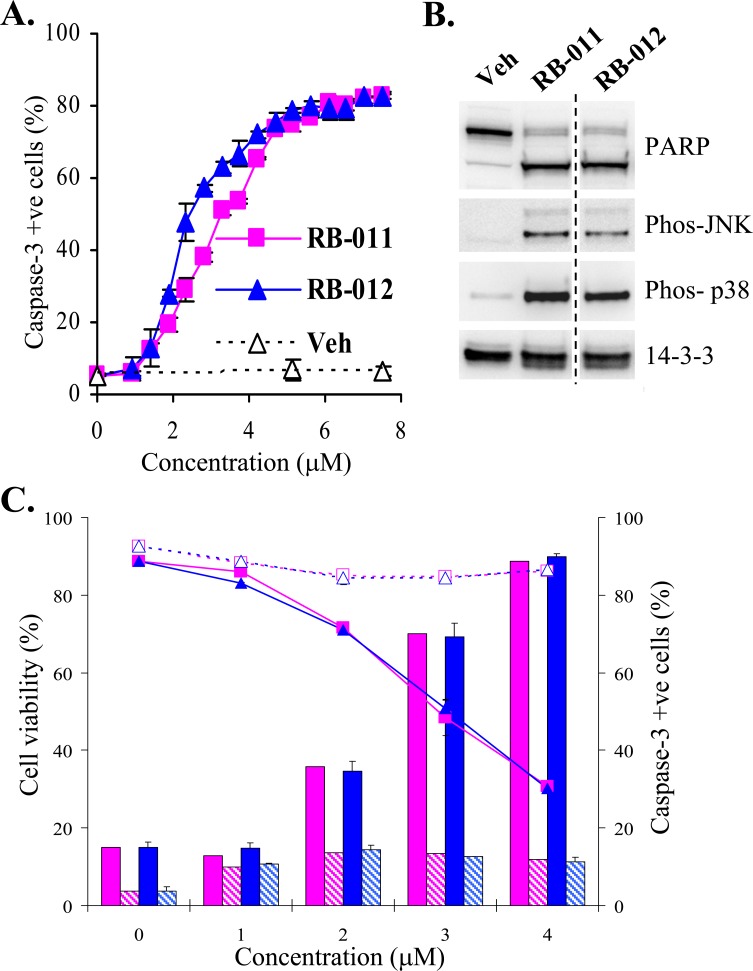
**A.** Dose response of caspase-3 activation (detected by flow cytometry using NucView^TM^) in Jurkat cells after 5 h treatment with RB-011 (pink squares), RB-012 (blue triangles) or vehicle (Veh). The error bars show the range of duplicate determinations. **B.** Immunoblotting of Jurkat lysates after 4 h treatment of cells with either vehicle or 7.5 μM RB-011 or RB-012. **C.** Effect of RB-011 (pink) and RB-012 (blue) on cell viability (shown by line graph) and caspase-3 activation (histograms) in parental Jurkat cells (solid lines and color) and Jurkat cells over-expressing Bcl-2 (dashed lines and hashed color) after 20 h treatment. The error bars show the range of duplicate determinations and the results are representative of several experiments.

The potency and apoptotic effects of RB-011 and -012 on cells were examined more closely. We determined the ED_50_ for apoptosis induction in Jurkat cells by assessing the activation of caspase-3 at 5 h, before any significant loss of cell viability (Figure 3B). RB-012 was slightly more potent than RB-011 at initiating apoptosis, with an ED_50_ of 2 μM compared with 3 μM for RB-011 (Figure 4A). Biochemical characterization of RB-treated Jurkat cells revealed that after 4 h of treatment PARP cleavage had occurred, consistent with the commitment to apoptosis (Figure 4B, upper panels). Using phospho-specific antibodies, we detected active stress-activated protein kinases, p38 and JNK, after 4 h of treatment with RB-011 or -012 (Figure 4B, second and third panel). Immunoblotting of 14-3-3 revealed a slightly lower molecular weight form of 14-3-3 after 4 h of RB-011 or -012 treatment (Figure 4B, bottom panel) probably associated with caspase cleavage as 14-3-3 proteins have previously been shown to be susceptible [28].

These analyses indicate that the activation of apoptosis in response to the RB compounds is rapid, and detectable by 4 h of treatment. Additionally and importantly, over-expression of Bcl-2 completely protected the Jurkat cells from the RB compounds (Figure 4C) as determined by analysis of viability and caspase activation after 20 h of treatment, confirming that the compounds initiate signaling upstream of mitochondrial-mediated apoptosis.

### The novel RB sphingomimetics cause rapid inhibition of PI3K-AKT and MAPK signaling

**Figure 5:**
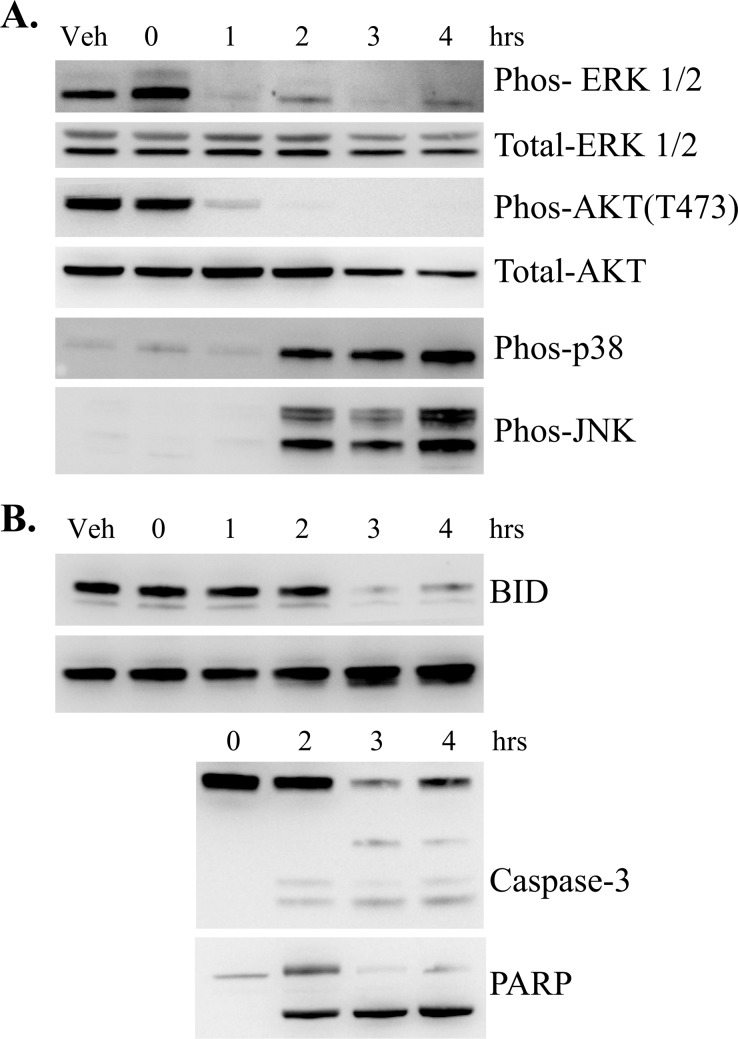
**A.** Immunoblotting analysis of signaling molecules (as shown) over time induced by 7.5 μM RB-012 treatment of Jurkat cells. **B.** Immunoblotting analysis of apoptotic signals (as shown) over time induced by 7.5 μM RB-012 treatment of Jurkat cells.

To characterize the signaling changes associated with RB treatment of Jurkat cells, we carried out time-course studies using RB-012 and prepared cytosolic extracts for immunoblotting. Strikingly, phospho-specific antibodies revealed rapid dephosphorylation of both ERK and AKT (within 1 h) upon RB-012 treatment (Figure 5A first and third panels), indicating down-regulation of the MAPK and PI3K signaling pathways respectively. Activation of SAPKs p38 and JNK was detected but not until 2 h post RB-012 treatment (Figure 5A fourth and fifth panels), after ERK and AKT inactivation.

In the same time-course studies, apoptotic markers were analyzed by immunoblotting. BID cleavage is associated with diverse apoptotic stimuli and has been shown to occur after mitochondrial permeability transition and apoptotic commitment via a caspase-3 mediated process in Jurkat cells [29]. We analyzed BID cleavage after RB-12 treatment and found that BID breakdown is detected at 3 h of RB-012 treatment (Figure 5B, first panel). Immunoblotting for 14-3-3 over the time-course revealed that the lower molecular weight form of 14-3-3 protein is detected at 2 h (Figure 5B, second panel), consistent with activation of caspase-3 (Figure 5B, third panel). Additionally, PARP cleavage was detectable at 2 h (Figure 5B, fourth panel). Thus commitment to apoptosis in response to RB-012 occurs within 2-3 h of treatment, after the initial effects on MAPK and PI3K signaling.

### RB sphingomimetics inhibit A549 lung cancer cell growth *in vitro* and *in vivo*

**Figure 6:**
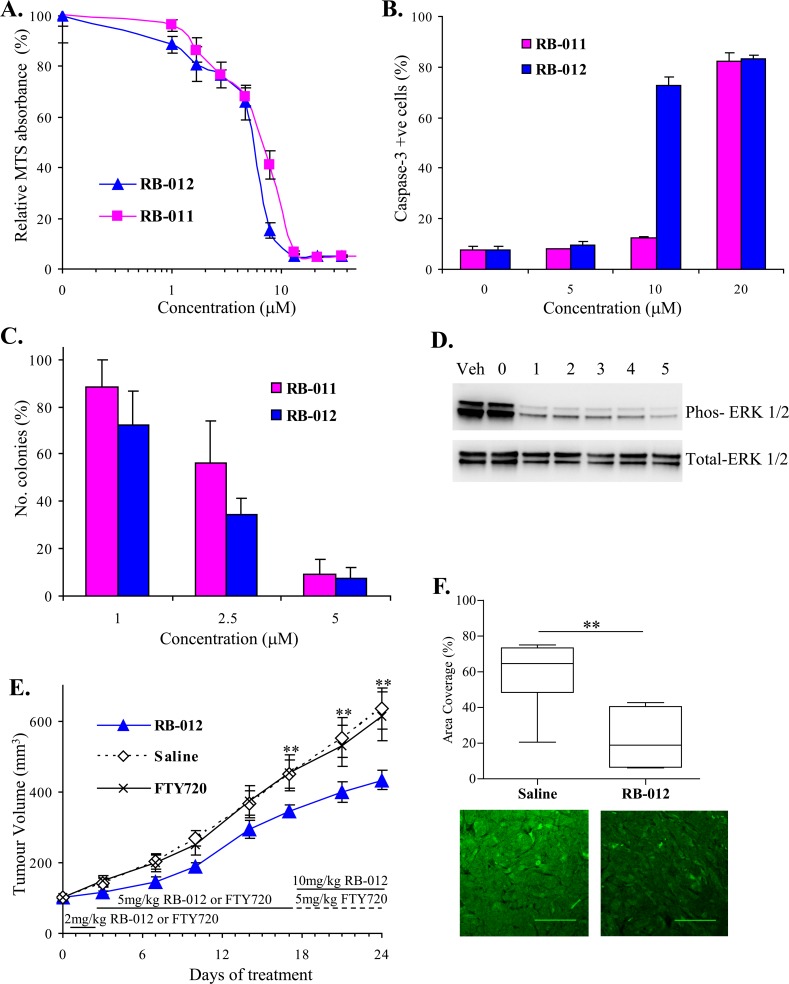
**A.** Viability of NSCLC cell line A549 is inhibited by RB-011 and RB-012 as determined by MTS assay after 48 h treatment. Error bars represent standard error of triplicate measurements. **B.** RB-011 and RB-012 induce caspase-3 activation at 48 h in A549 cells. The error bars show the range of duplicate determinations: and the results are representative of several experiments. **C.** Effect of RB-011 and -012 on A549 colony growth in soft agar. Results are expressed relative to colony numbers in untreated controls. **D.** Immunoblotting analysis of phospho-ERK over time induced by 25 μM RB-012 treatment of A549 cells. **E.** Growth of A549 xenograft in BALB/c nude mice is retarded by administration of RB-012. RB-012, saline or FTY720 was administered daily to mice bearing A549 tumors by intraperitoneal injection using the dosing regime shown. All experimental data are shown as the mean ± SEM. ** indicates *P* < 0.05. **F.** RB-012 induces down-regulation of MAPK signaling in A549 xenografts. Tumors were excised at the end of the study and analyzed by immunofluorescence for phospho-ERK. Area coverage analysis is represented by a box and whisker plot. Statistical significance was assessed using the Mann-Whitney test and Dunnet's post hoc test, ** indicates *P* < 0.05 for *N* = 7 samples with multiple fields analyzed. Representative images of phospho-ERK immunofluorescence are shown below, scale bar – 100 μm.

RB-011 and -012 exhibit apoptotic activity on Jurkat cells. Their effects are mediated at least in part by disruption of functional 14-3-3 dimers and inactivation of AKT and MAPK signaling pathways. These characteristics are desirable for a new anti-cancer therapy. We therefore sought to validate these compounds on human cancer cells where 14-3-3 over-expression has been implicated in tumor aggression. Of the multiple cancer types where 14-3-3 over-expression has been detected, non-small cell lung cancer (NSCLC) has been identified as a cancer in which the degree of 14-3-3ζ over-expression correlates strongly with poor patient survival and disease severity [4]. We therefore assessed the effect of the RB-011 and -012 molecules on the NSCLC line A549 (Figure 6).

First, we performed survival assays using MTS and found that the RB compounds reduced cell viability in a dose-dependent manner (Figure 6A). As with the Jurkat cells, RB-012 was slightly more potent than RB-011 with an IC_50_ of 5.5 μM compared with 7 μM for RB-011 (Figure 6A). We assessed the ability of the RB compounds to induce A549 cells apoptosis by monitoring caspase-3 activation. Both compounds induced caspase-3 activation with RB-012 showing greater potency at 10 μM (Figure 6B). The compounds were then tested for their effect on A549 colony formation in soft agar, a measure of neoplastic growth. Both compounds reduced colony formation in a dose-dependent manner with RB-012 again showing slightly greater potency (Figure 6C).

A549 cells harbor oncogenic K-Ras and consequently constitutive MAPK signaling is characteristic of this cell line. We analyzed the effect of RB-012 on MAPK signaling in the A549 cells *in vitro* and observed a rapid reduction in phospho-ERK in response to RB-012 (Figure 6D). Thus given RB-012's activity *in vitro*, we then studied its effects *in vivo*.

To assess any potential toxicity, BALB/c nude mice were administered either RB-012 or FTY720, delivered in saline (0.9%), daily by i.p. injection at 5mg/kg or 10mg/kg body weight, doses previously reported for FTY720 administration [21]. Over a course of 28 days there were no adverse effects associated with RB-012 treatment with the exception of apparent pain and abdominal cramping in the mice immediately after injection which subsided within 10-20 min. Similar effects were also observed with 5mg/kg/day FTY720 treatment. Importantly there was no pathology associated with the abdominal cramping suggesting a physiological response and over time the mice developed a tolerance to this effect. The body weight of RB-012 treated mice was unaltered over the course of the toxicity study and histology of tissues (liver, lung, kidney, heart and spleen) collected during the treatment course was unaffected (supplementary Figure 2A) indicating that the RB-012 had no major toxic effects. Additionally, bone marrow samples from RB-012 treated mice exhibited no alterations indicating no changes in steady state haemopoiesis (supplementary Figure 2B). In the same toxicity study we found that FTY720 dosed at 10mg/kg body weight was poorly tolerated and caused cardiac arrhythmia with one unexplained fatality. Toxicity has previously been noted with i.p. injection of FTY720 at 10mg/kg/day [30] and we were therefore unable to use this dose of FTY720 in the subsequent xenograft study.

To assess the effect of RB-012 on human lung cancer growth *in vivo,* A549 cells were implanted subcutaneously on the flanks of BALB/c nude mice and tumors were allowed to grow until they had reached a volume of 100 mm^3^. RB-012, FTY720 or saline was then administered daily to assess the effect on tumor growth. Owing to the physiological abdominal cramping response observed in the toxicity study, we were required on ethical grounds to use a dose escalation regime for administering both RB-012 and FTY720 to allow the mice to develop tolerance. Initially, dosing was limited to 2mg/kg body weight for two days and then increased to 5mg/kg daily for two weeks which minimized the cramping. Tumor volume was monitored twice a week and after two weeks of dosing at 5mg/kg body weight, mice administered RB-012 had tumors that were 20% smaller than mice receiving saline (Figure 6E). Interestingly, mice receiving FTY720 showed no reduction in tumor size (Figure 6E) in contrast to a previous report employing A549 xenografts [31] albeit in a different mouse strain (Balb/c SCID) and using a different route of FTY720 administration (by oral gavage).

As RB-012 was better tolerated than FTY720, the dosing of RB-012 treated mice was increased to 10mg/kg body weight for a further week with no adverse effects on the mice. The tumors on the RB-012-treated mice continued to grow more slowly than those on saline treated mice and at the end of the treatment course were 30% smaller than those of saline-treated mice (Figure 6E). Thus RB-012 effectively reduced A549 tumor growth whereas FTY720 was ineffective in this study.

To provide evidence that the anti-cancer effect of RB-012 in the A549 xenograft study involved disruption of dimeric 14-3-3, we analyzed the excised tumor tissue for MAPK activity. Quantitative immunofluorescence analysis showed that tumors treated with RB-012 had significantly less phospho-ERK than saline-treated tumors (Figure 6F), consistent with the *in vitro* results on A549 cells (Figure 6D). Thus RB-012 effectively reduces A549 xenograft growth by a mechanism involving disruption of the oncogenic signaling in these cells, supporting the notion that RB-012 reduces tumor growth by disruption of dimeric 14-3-3 function.

## DISCUSSION

The widespread over-expression of 14-3-3 proteins in human tumors has highlighted the significance of 14-3-3 proteins in cancer development [4-8]. Increased 14-3-3 expression provides cancer cells with enhanced protection against apoptotic mediators making 14-3-3 proteins an attractive target for anti-cancer drug development. Several groups have identified molecules with the ability to block the binding of 14-3-3 proteins to client proteins [13, 14, 32]. Without exception these molecules compete with client proteins for binding in the amphipathic groove [32]. We have developed a novel approach that exploits discrete sphingolipid binding site(s) on 14-3-3 that when occupied, causes dimer disruption and loss of function [15].

We have now identified *N*-alkylated trimethylammonium (TMA) molecules as modulators of 14-3-3 *in vitro* with a corresponding capacity to induce apoptosis of Jurkat cells (Figure 1). From a chemical series of trimethylammonium compounds we determined that the length of the alkyl chain is an important factor in determining the effect on 14-3-3 modulation and Jurkat cell apoptosis. Long-chain TMAs have the greatest potency both in *in vitro* 14-3-3 phosphorylation assay and inducing Jurkat cell apoptosis (Figure 1). We have demonstrated that at low micromolar concentrations (below the CMC), CTAB modulates 14-3-3ζ, allowing phosphorylation by PKA at Ser58 in the dimer interface, in an analogous fashion to sphingosine and its analogues [15]. Additionally, the long-chain TMAs induce apoptosis in Jurkat cells at concentrations well below CMC via the mitochondrial pathway (Figure 2). These data support the notion that long-chain TMAs induce apoptosis by interfering with dimeric 14-3-3 in the cell.

CTAB was previously identified in a high-throughput screen for anti-cancer agents as being able to inhibit the growth of a panel of head and neck cancer (HNC) cell lines and reduce mitochondrial membrane potential (ΔΨ_M_) but the mechanism of action was unknown [33]. Consistent with our results, the apoptotic effect of CTAB was related to the length of the alkyl group [33]. Furthermore, CTAB administration at 5 mg/kg retarded the growth of HNC xenografts *in vivo* [33]. 14-3-3ζ over-expression has been reported in HNCs and correlates with poor patient prognosis [5] and knock-down of 14-3-3ζ in HNC cell lines by RNAi inhibited cell growth and increased apoptosis in response to chemotherapy [10]. Our results strongly suggest that the effects of CTAB on HNC lines are attributable to 14-3-3 dimer disruption.

CTAB has also been assessed for anti-cancer activity in the National Cancer Institute Developmental Therapeutics Program (www.dtp.nci.nih.gov) in mouse cancer models of lymphocytic leukemia (P388 and L1210) and melanoma (B16). A modest therapeutic effect was seen in the P388 model at low doses, at 3 and 6 mg/kg body weight per day i.p. 20 % more of the mice survived to 30 days of treatment compared with untreated controls. At high dose (>10 mg/kg body weight) CTAB was toxic (after 5 days treatment, data extracted from DTP at NCI) and given its strong surfactant properties is not suitable as a drug candidate. However, CTAB's ability to disrupt 14-3-3 dimers, allowing phosphorylation in the dimer interface, coincident with its ability to induce mitochondrial apoptosis and experimental anti-cancer activity, provide initial validation for our strategy to identify compounds with 14-3-3-dimer disrupting activity as potential anti-cancer agents.

In the present study we designed a series of new compounds that combine the quaternary ammonium group of the alkylated TMA molecules with the backbone of FTY720. The compounds exhibit varying ability to render 14-3-3 phosphorylatable *in vitro* coinciding with their ability to induce apoptosis of Jurkat cells (Figure 3). In particular RB-011 and RB-012 have a potent ability to induce apoptosis of Jurkat cells within 4 h of treatment and have ED_50_ values in the low micromolar range (Figure 4). Time-course studies revealed rapid inactivation of ERK and AKT signaling within 1 h of treatment of Jurkat cells with RB-012, followed by activation of SAPKs, JNK and p38 by 2 h (Figure 5A). The apoptotic cascade then proceeds with caspase-3 processing, PARP and BID cleavage (Figure 5B). These are important findings that reveal the cellular effects of RB-012 occur in a time-dependent manner with primary effects on cell signaling occurring prior to apoptotic commitment.

14-3-3 proteins are known to play a key role in integrating survival signaling within the cell [1-2]. When mutant forms of 14-3-3 that are non-competent for phospho-client binding are expressed in cells they heterodimerise with endogenous isoforms and act as functionally monomeric proteins, causing disruption of MAPK signaling and activation of SAPK [34, 35]. These effects phenocopy what we have observed with RB-012, and match the known differential regulation of upstream MAPK Raf-1 [36] and SAPKK, ASK-1 [37] and MEKK2 [38] by dimeric 14-3-3 proteins. Additionally, recent studies have confirmed that 14-3-3 proteins are required for PI3K activity [39]. The downstream inactivation of AKT signaling in response to RB-012 is therefore entirely consistent with 14-3-3's role in PI3K function. Our studies reveal that targeting 14-3-3 proteins using our sphingomimetic approach provides a potent means to disrupt functional 14-3-3 dimers which is desirable for an effective anti-cancer drug.

Several isoforms of 14-3-3 have been associated with the transformed phenotype, both experimentally; over-expression of 14-3-3γ, β and ζ have been shown to cause cellular transformation [9, 40, 41], and in clinical samples (ζ, β, ε, γ, η and τ) [4-8]. However, the 14-3-3ζ isoform is the most commonly up-regulated in cancer [4-8]. This may be due to a specific role for the zeta isoform although to date none are known. More likely, the specific regulation of 14-3-3ζ expression by a micro-RNA (miR-) 451 may be the underlying cause [42]. The negative regulation of 14-3-3ζ by miR-451 has been elegantly demonstrated in breast cancer cells where it was shown that tamoxifen treatment leads to loss of miR-451 expression and up-regulation of 14-3-3ζ, correlating with increased disease severity [42]. Intriguingly, miR-451 expression has also been shown to be low in NSCLC tissue compared with non-cancerous lung tissue, and up-regulation of miR-451 in A549 cells reduced cell growth and increased the cells susceptibility to cisplatin [43]. These results mirror the over-expression 14-3-3ζ seen in NSCLC tissue which correlates with disease severity, and similarly targeted knock-down of 14-3-3ζ using RNAi in A549 cells also increased the cells sensitivity to cisplatin [4]. Thus the reciprocal expression of miR-451 and 14-3-3ζ may be important prognostic markers for disease severity and cancer progression in NSCLC.

As 14-3-3ζ expression is closely linked with NSCLC disease, we tested RB-011 and RB-012 for anti-cancer effects on NSCLC A549 cells. We found that these compounds inhibited A549 cell survival and colony formation in soft-agar and induced apoptosis (Figure 6). 14-3-3ζ knock-down in A549 cells has previously been shown to reduce colony formation and increase anoikis (apoptosis induced by loss of cell adhesion) [11]. We also demonstrated the therapeutic potential of RB-012 in an *in vivo* A549-xenograft model (Figure 6) and showed that RB-012 reduces tumor growth by up to 30% over a three week course of treatment. The compound was well tolerated with fewer side effects than FTY720, which did not elicit a reduction in tumor growth. FTY720 has previously been demonstrated to reduce tumor growth in other experimental cancer models [21] but at higher doses (10 mg/kg/day), suggesting that the RB-012 is more potent. FTY720 is readily converted to a phospho-form by an endogenous sphingosine kinase (SK), and in this form acts as a sphingosine-1-phosphate analogue which mediates the drug's immunosuppressive action. RB-012 cannot be phosphorylated as it lacks a phosphate accepting hydroxyl group and unlike FTY720, does not affect SK activity [22]. Therefore compared to FTY720, RB-012 has a more selective anti-cancer action. Compared with other 14-3-3-directed small molecules [13, 14 & 32], the RB compounds shown here are non-competitive in that they do not compete with endogenous phospho-clients for binding in the amphipathic groove. These data provide valuable proof-of-principle for our 14-3-3 dimer disruption approach to cancer drug discovery.

## MATERIALS AND METHODS

### Compounds

*N-*alkylated tri-methyl ammonium (TMA) compounds were purchased from Sigma. RB compounds were generated as mesylate salts and sodium mesylate was used in all relevant vehicle treatments.

RB-011, -012 and -015 were synthesized as described previously [22]. RB-066, RB-067, and RB-068 were prepared by the following procedures and characterized by ^1^H and ^13^C NMR spectroscopy and electrospray ionization high-resolution mass spectrometry (ESI-HRMS).

#### 4-Methyl-4-(4-octylphenethyl)morpholin-4-ium methanesulfonate (RB-066)

To a solution of 4-octylphenethyl methanesulfonate (10 mg, 0.032 mmol) in 3 mL of acetonitrile was added *N*-methylmorpholine (70.4 μL, 0.64 mmol). The reaction mixture was stirred at 50°C for 2 d and concentrated. The residue was washed with hexane to give 7 mg (52%) of RB-066 as a yellow liquid; ^1^H NMR (400 MHz, CDCl_3_) δ 0.88 (t, *J* = 6.9 Hz, 3H), 1.26–1.29 (m, 10H), 1.57 (t, *J* = 7.9 Hz, 3H), 2.56 (t, *J* = 7.7 Hz, 3H), 2.76 (s, 3H), 3.08–3.12 (m, 2H), 3.46 (s, 3H), 3.49–3.53 (m, 2H), 3.67–3.70 (m, 2H), 3.81–3.85 (m, 2H), 3.92–4.00 (m, 4H), 7.14 (d, *J* = 8.0 Hz, 2H), 7.21 (d, *J* = 8.0 Hz, 2H); ^13^C NMR (100 MHz, CDCl_3_) δ 14.1, 22.7, 28.1, 29.3 (2C), 29.4, 29.5, 31.5, 31.9, 35.6, 39.6, 42.8, 47.7, 60.7, 65.7, 128.9, 129.3, 136.1, 142.0; ESI-HRMS (M + H)^+^ *m*/*z* calcd for C_21_H_36_NO 318.2797, found 318.2796.

#### 1-Methyl-1-(4-octylphenethyl)-4-oxopiperidinium methanesulfonate (RB-067)

To a solution of 4-octylphenethyl methanesulfonate (10 mg, 0.032 mmol) in 3 mL of acetonitrile was added 1-methyl-4-piperidone (74.7 μL, 0.64 mmol). The reaction mixture was stirred at 50°C for 2 d and concentrated. The residue was washed with hexane to give 8 mg (59%) of RB-067 as a yellow liquid; ^1^H NMR (400 MHz, CDCl_3_) δ 0.81 (t, *J* = 6.7 Hz, 3H), 1.02 (s, 9H), 1.12–1.25 (m, 24H), 1.32–1.76 (m, 2H), 3.47–3.51 (m, 1H), 3.68–3.82 (m, 3H), 4.20–4.23 (m, 1H), 4.47 (dd, *J* = 11.2, 19.6 Hz, 2H), 7.16–7.20 (m, 4H), 7.26–7.40 (m, 6H), 7.57–7.62 (m, 5H); ^13^C NMR (100 MHz, CDCl_3_) δ 14.1, 19.1, 22.7, 25.5, 26.8, 26.8 (2C), 29.4 (2C), 29.5 (2C), 29.6, 29.7 (2C), 31.9, 55.6, 63.9, 65.0, 72.1, 79.9, 127.7, 127.8 (3C), 127.9, 128.4, 130.0, 135.6 (3C), 135.7, 137.9; ESI-HRMS (M + H)^+^ *m*/*z* calcd for C_22_H_36_NO 330.2797, found 330.2791.

#### 8-Methyl-8-(4-octylphenethyl)-1,4-dioxa-8-azoniaspiro[4.5]decane methanesulfonate (RB-068)

To a solution of 4-octylphenethyl methanesulfonate (10 mg, 0.032 mmol) in 3 mL of acetonitrile was added 8-methyl-1,4-dioxa-8-azaspiro[4.5]decane (100 mg, 0.64 mmol). The reaction mixture was stirred at 50°C for 24 h and concentrated. The residue was washed with hexane to give 13 mg (85%) of RB-068 as a yellow liquid; ^1^H NMR (400 MHz, CDCl_3_) δ 0.88 (t, *J* = 6.8 Hz, 3H), 1.26–1.29 (m, 10H), 1.56 (t, *J* = 7.2 Hz, 2H), 1.90–1.94 (m, 2H), 2.06–2.11 (m, 2H), 2.54 (t, *J* = 7.7 Hz, 2H), 2.73 (s, 3H), 3.08–3.21 (m, 2H), 3.39(s, 3H), 3.57–3.61 (m, 2H), 3.72–3.76(m, 4H), 3.94–4.00 (m, 4H), 7.12 (d, *J* = 8.0 Hz, 2H), 7.23 (d, *J* = 8.0 Hz, 2H); ^13^C NMR (100 MHz, CDCl_3_) δ 14.1, 22.7, 27.0, 28.6, 29.3(2C), 29.5, 30.0, 31.5, 31.6, 31.9, 35.5, 39.6, 59.8, 64.9, 65.0, 103.3, 128.9, 129.2, 131.9, 142.5; ESI-HRMS (M + H)^+^ *m*/*z* calcd for C_24_H_40_NO_2_ 374.3059, found 374.3055.

### 14-3-3 phosphorylation assays

Substrate 14-3-3 (0.5 μg of purified recombinant 14-3-3) was added to 15 μl of reaction mixture comprising 0.2U of the PKA catalytic subunit, in the presence or absence of compounds (delivered in 0.1% v/v ethanol) in PKA reaction buffer (10 mM Tris-HCl pH 7.4, 15 mM MgCl_2_, 3 mM DTT containing 25 μM ATP and 0.3 μCi [^32^P] γ-ATP). Reactions were incubated at 37°C for 15 min. After incubation, reactions were separated on 12.5% SDS-PAGE and Coomassie stained. 14-3-3 phosphorylation was analyzed using a Typhoon Phosphorimager and quantified using Molecular Dynamics Image Q 5.2 software.

### PKA activity assays

Reactions were essentially identical to 14-3-3 phosphorylation assays except that 50 μM kemptide substrate was added in place of 14-3-3 protein. After incubation at 37°C for 15 minutes the reactions were spotted onto phosphocellulose filters (Whatman P81). Filters were washed 5 times in 0.75% phosphoric acid and once in acetone before liquid scintillation counting.

### Cell lines and culture

Jurkat E6.1 cells were obtained from the ATCC and verified by short tandem repeat (STR) analysis in December 2014. The A549 cell line was purchased from ECACC in April 2012 (which also performs STR verification) and used within 6 months of resuscitation in the studies presented here. Jurkat cells were routinely cultured in RPMI with 10% FBS and A549 cells in DMEM with 10% FBS at 37ºC with 5% CO_2_. Jurkat Bcl-2 cells were generated by lentiviral transduction using a third generation lentiviral construct as described previously [15] containing a Bcl-2α-IRES-IL2Rα encoding cassette. Transduced cells were FACS sorted for expression of IL2Rα using anti-CD25-PE (BD Pharmingen, #555433) (Supplementary Figure 1A) to enrich for Bcl-2 over-expressing cells, and protein expression was further confirmed by immunoblotting with anti-Bcl-2 antibody (BD Transduction Laboratories, #610538) (Supplementary Figure 1B).

### Apoptosis assays: TMRE, Caspase 3 and Annexin V

Jurkat cells were routinely set up in apoptosis assays at 2 × 10^5^/ml in RPMI with 0.5% FBS. After treatment, cells were stained either with 200 nM TMRE, 5 μl/ml NucView^TM^ (Biotium), or Annexin V-FITC (Roche) for 15 min prior to analysis by flow cytometry. Forward- and side-scatter properties were used to exclude debris and a ‘viable’ gate corresponding to the intact PI negative cell population was used for fluorescence analysis. For A549, cells at 90% confluency were treated with the compounds for 48h in DMEM with 0.5% FBS. After treatment, cells were released with trypsin and then stained with NucView^TM^prior to analysis by flow cytometry.

### Assessment of A549 cell viability by MTS assay

A549 cells (2500/well) were plated in 96-well trays in DMEM with 0.5% FBS and cultured at 37ºC with 5% CO_2_ overnight. The following day RB compounds were added and the cells were incubated for a further 48 h prior to removal of the medium and replacement with MTS reagent (Promega) diluted 1:6 in Dulbecco's PBS. The cells were incubated at 37ºC for a further 4 h and the conversion of MTS to the colored formazan compound was determined by measurement of absorbance at 490 nM.

### A549 colony assay

A549 cells (7500) were plated in 0.33% low-melting point agarose in DMEM with 10 % FBS with and without RB compounds over a 0.5% low-melting point agarose base. Cells were incubated at 37ºC with 5% CO_2_ for 14 days and then colonies were photographed and analyzed using Image J.

### Immunoblotting

Jurkat cells were treated at 5 × 10^5^/ml in RPMI with 0.5% FBS as detailed. Jurkat cells were harvested by centrifugation at 1500 g for 5 min and washed with PBS prior to lysis in homogenization buffer (20 mM Tris-HCl pH 7.4, 0.5 mM EDTA, 0.5 mM EGTA, 5 % glycerol, protease inhibitor (Roche), 4 mM NaF, 2 mM sodium vanadate, 10 mM β-glycerophosphate, 1 mM sodium pyrophosphate, 1 mM sodium molybdate) for 15 minutes on ice followed by three rounds of freeze-thawing. For treatment A549 cells were plated in 10 cm dishes and treated when they reached approximately 75 % confluence in DMEM with 0.5% FBS. Cells were scraped from the dish and collected by centrifugation at 1500 g and washed with PBS prior to lysis in homogenization buffer as detailed above.

All lysates were clarified at 13,000 rpm for 20 minutes at 4°C and protein concentration was determined using the BCA assay (Pierce). Thirty to forty μg of lysate were run on Criterion-XT 4-12 % Bis-Tris gels (Bio-Rad), followed by transfer onto nitrocellulose. The filters were blocked for 30 min at room temperature in TNT buffer (10 mM Tris-HCl pH 8.0, 150 mM NaCl, 0.05 % Tween-20) containing blocking buffer (Roche), prior to incubation with antibodies over night. Antibodies used were from Cell Signaling Technology; phospho-p38 MAPK (#4571), phospho-SAPK/JNK (81E11), phospho- ERK (#9101), phospho-AKT S473 (#9271), ERK (#9102), AKT (#9272), BID (#2002), caspase-3 (#9602), PARP (#9542), and Santa Cruz Biotechnology; pan 14-3-3 K19 (sc-629). After antibody binding, filters were washed in TNT for 1h at room temperature, incubated with 1/12,500 anti-rabbit or anti-mouse HRP secondary antibody (Pierce) for 1h at room temperature and finally washed for 1 hr with TNT before incubation with Clarity western ECL reagent (Bio-Rad). Filters were exposed on a LAS 4000 imager. Blots were analyzed using Multi-gauge/Colony software FUJI FILM.

### *In vivo* toxicity and xenograft studies

All experimental procedures involving animals were conducted in accordance with the NHMRC Australian Code for the Care and Use of Animals for Scientific Purposes and with approval by the institutional animal ethics committee. BALB/c nude mice (Nu/Nu, female, 5-6 weeks old) were purchased from the Animal Resources Centre (Perth, WA) and maintained under pathogen-free conditions.

Toxicity studies were carried out with both RB-012 and FTY720 (in 0.9% saline) at 5 and 10 mg/kg body weight, administered daily by intraperitoneal injection and mice were monitored over a 28 day treatment course. Five mice were used per group for RB-012 treatment and three mice per group for FTY720 treatment.

For xenograft studies, A549 cells (5 × 10^6^) in 100 μl of PBS were injected subcutaneously into the flanks of the mice and the resulting tumors were measured using digital calipers. The tumor volume was calculated using the following formula: Volume = (larger diameter) × (small diameter)^2^/2. Once the tumors had reached 100 mm^3^, mice were divided into groups of 9 and to each group was administered with either RB-012, FTY720 (in 0.9% saline) or saline by intraperitoneal injection daily. Initially, RB-012 and FTY720 were administered at 2 mg/kg body weight for two days and then the dose was increased to 5 mg/kg body weight for two weeks, after which RB-012 was increased to 10 mg/kg body weight but FTY720 was maintained at 5 mg/kg body weight. During this dosing regime tumors were monitored twice a week. Differences between samples were analyzed using 2-way ANOVA and statistical significance was accepted at P < 0.05.

### Tissue preparation and analysis

At the end of the treatment courses, mice were euthanized and tissues were collected and fixed in neutral-buffered formalin for 12 hrs at 4°C. Tissues were processed by embedding in paraffin and cutting into 4 μm sections. Sections were dewaxed and rehydrated and the antigen retrieved by boiling for 20 minutes in 10 mM citrate buffer (pH 6.0) under pressure (65 kPa above atmospheric pressure). Sections were blocked with 10 % goat serum in PBS (pH 7.4) solution for 30 minutes and incubated with rabbit anti-phospho-ERK (1:100 diluted; CST #9101) at 4°C overnight. Sections were washed three times in PBS containing 0.1 % Tween and incubated with anti-rabbit IgG conjugated to Alexa Fluor-488 (1:400 diluted; Invitrogen) at room temperature for one hour. Sections were washed as before and mounted in vectashield hard-set mounting medium (Vector) containing DAPI and imaged using a LSM 710 two-photon microscope (Zeiss). Images were analyzed using ImageJ software (NIH) to calculate percentage area coverage by fluorescence signal per image using a binary converted image based on a single manually determined threshold value applied across all images (as previously described) [23, 24]. Results are expressed as medians with ranges and quartiles across all data sets.

## SUPPLEMENTARY MATERIAL FIGURE


