# Supplementary material for: Destabilisation of dimeric 14-3-3 proteins as a novel approach to anti-cancer therapeutics
Source: Oncotarget. 2015 May 4;6(16):14522–36. doi: 10.18632/oncotarget.3995 (PMC4546484; doi:10.18632/oncotarget.3995)
Supplement: Supplementary file 1 [file oncotarget-06-14522-s001.pdf]

Destabilisation of dimeric 14-3-3 proteins as a novel approach to anti-cancer therapeutics

Supplementary Material

Supplementary Fig.1

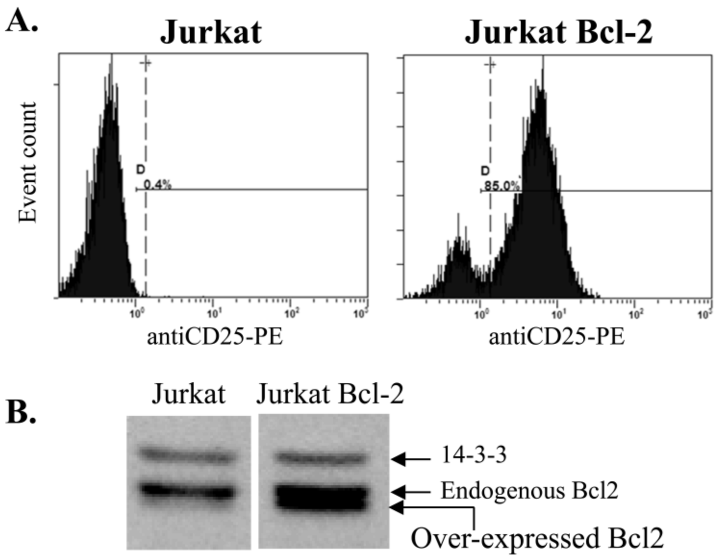

Supplementary Figure 1 Generation of Jurkat Bcl-2 cells by lentiviral transduction. **A.** Cells transduced with lentivirus containing a Bcl-2α-IRES-IL2Rα encoding cassette were fluorescence activated cell sorted for expression of IL2Rα using anti-CD25-PE to enrich for Bcl-2 over-expressing cells. **B.** Protein expression was further confirmed by immunoblotting for Bcl-2. 14-3-3 immunoblotting is shown as a loading control.

Supplementary Fig.2

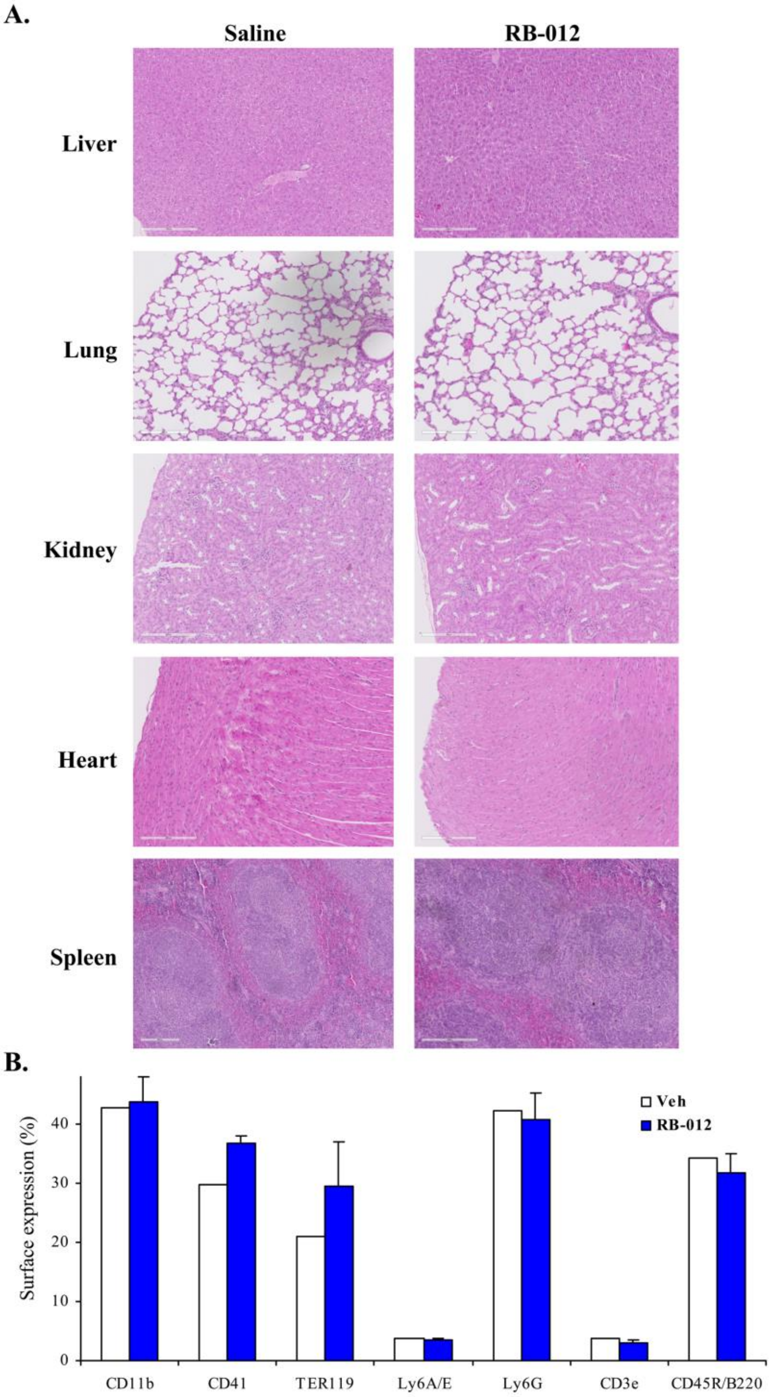

Supplementary Figure 2 A. Hematoxylin and eosin staining of tissues from mice treated with saline or 10mg/kg/day RB-012 for 14 days. Scale bar represents 200 μm. **B.** Flow analysis of bone marrow samples taken from RB-012 (10mg/kg/day) or saline treated mice after 14 days. Positive staining for haemopoietic lineages is shown.
